# Specificity of Multi-Modal Aphid Defenses against Two Rival Parasitoids

**DOI:** 10.1371/journal.pone.0154670

**Published:** 2016-05-02

**Authors:** Adam J. Martinez, Kyungsun L. Kim, Jason P. Harmon, Kerry M. Oliver

**Affiliations:** 1 Department of Entomology, University of Georgia, Athens, Georgia, United States of America; 2 Department of Entomology, North Dakota State University, Fargo, North Dakota, United States of America; University of Basilicata, ITALY

## Abstract

Insects are often attacked by multiple natural enemies, imposing dynamic selective pressures for the development and maintenance of enemy-specific resistance. Pea aphids (*Acyrthosiphon pisum*) have emerged as models for the study of variation in resistance against natural enemies, including parasitoid wasps. Internal defenses against their most common parasitoid wasp, *Aphidius ervi*, are sourced through two known mechanisms– 1) endogenously encoded resistance or 2) infection with the heritable bacterial symbiont, *Hamiltonella defensa*. Levels of resistance can range from nearly 0–100% against *A*. *ervi* but varies based on aphid genotype and the strain of toxin-encoding bacteriophage (called APSE) carried by *Hamiltonella*. Previously, other parasitoid wasps were found to commonly attack this host, but North American introductions of *A*. *ervi* have apparently displaced all other parasitoids except *Praon pequodorum*, a related aphidiine braconid wasp, which is still found attacking this host in natural populations. To explain *P*. *pequodorum’s* persistence, multiple studies have compared direct competition between both wasps, but have not examined specificity of host defenses as an indirectly mediating factor. Using an array of experimental aphid lines, we first examined whether aphid defenses varied in effectiveness toward either wasp species. Expectedly, both types of aphid defenses were effective against *A*. *ervi*, but unexpectedly, were completely ineffective against *P*. *pequodorum*. Further examination showed that *P*. *pequodorum* wasps suffered no consistent fitness costs from developing in even highly ‘resistant’ aphids. Comparison of both wasps’ egg-larval development revealed that *P*. *pequodorum*’s eggs have thicker chorions and hatch *two* days later than *A*. *erv*i’s, likely explaining their differing abilities to overcome aphid defenses. Overall, our results indicate that aphids resistant to *A*. *ervi* may serve as reservoirs for *P*. *pequodorum*, hence contributing to its persistence in field populations. We find that specificity of host defenses and defensive symbiont infections, may have important roles in influencing enemy compositions by indirectly mediating the interactions and abundance of rival natural enemies.

## Introduction

Host-parasitoid interactions are ubiquitous, consisting of a parasite which kills its host as a prerequisite for completing development [1], thus imposing strong selective pressures on both parties to survive the antagonistic interaction [2]. Insect hosts often deploy the cellular arm of their innate immune system to encapsulate and asphyxiate internally developing parasitoids [3–5] or engage in defensive mutualisms with microbial symbionts for protection [6]. Parasitoids, in turn, have evolved specific tactics (e.g. venom, teratocytes, polydnaviruses) to overcome host- or symbiont-derived defenses, and commandeer resources, in an effort to create a suitable environment for wasp development [7–10]. The evolution of host resistance and wasp counter-resistance can result in the specialization of traits that mediate host-parasitoid interactions [11]. Given that insect hosts are often attacked by multiple parasitoid species, e.g. [12–17], hosts may vary in resistance to particular natural enemies, e.g. [13, 18]. Such differences in resistance may occur locally or globally and, especially when multiple parasitoids are present, impact or depend on the dynamics of competing parasitoids, ultimately influencing other factors such as composition of natural enemies, enemy and host abundance, and evolutionary history of interacting parasitoids and their respective host species.

Aphids have emerged as important models for the study of variation in resistance to parasitoids, including defensive symbiosis [19]. The pea aphid, *Acyrthosiphon pisum* (Hemiptera: Aphididae)—*Aphidius ervi* (Hymenoptera: Braconidae: Aphidiinae) interaction is particularly well-studied. The pea aphid is a polyphagous pest of herbaceous legumes such as alfalfa and clover and its dominant parasitoid in North America is *A*. *ervi* [20–23]. Pea aphids, however, maintain high variation (0 to nearly 100%) in their susceptibility to *A*. *ervi* [24–26].

Given that pea aphids have a weak cellular encapsulation response to parasitoids [27–29] it was recently assumed that the bulk of their variation in resistance was owed to infection with the defensive bacterial endosymbiont, *Hamiltonella defensa* [26, 30, 31]. More recent work, however, indicates that strong resistance can be derived from endogenous aphid-encoded mechanisms as well [25]. Little is known about how innate or symbiont-based aphid defenses harm wasps, however, toxin-encoding bacteriophages, called APSEs, are required to infect *H*. *defensa* to produce the defensive mutualism [26]. There are multiple strains of *H*. *defensa*-APSE, but *H*. *defensa* containing either APSE2 (carries *cdtB* toxin allele) or APSE3 (carries *YDp* toxin allele) are found most commonly among North American pea aphids and are associated with moderate to high protection, respectively, against parasitism by *A*. *ervi* [8, 9, 32]. Based on previous developmental assays, APSE3 strains appear to kill developing *A*. *ervi* shortly after egg hatching, while mortality caused by APSE2 strains is more variable, but generally occurs later in wasp development [8].

**Fig 1:**
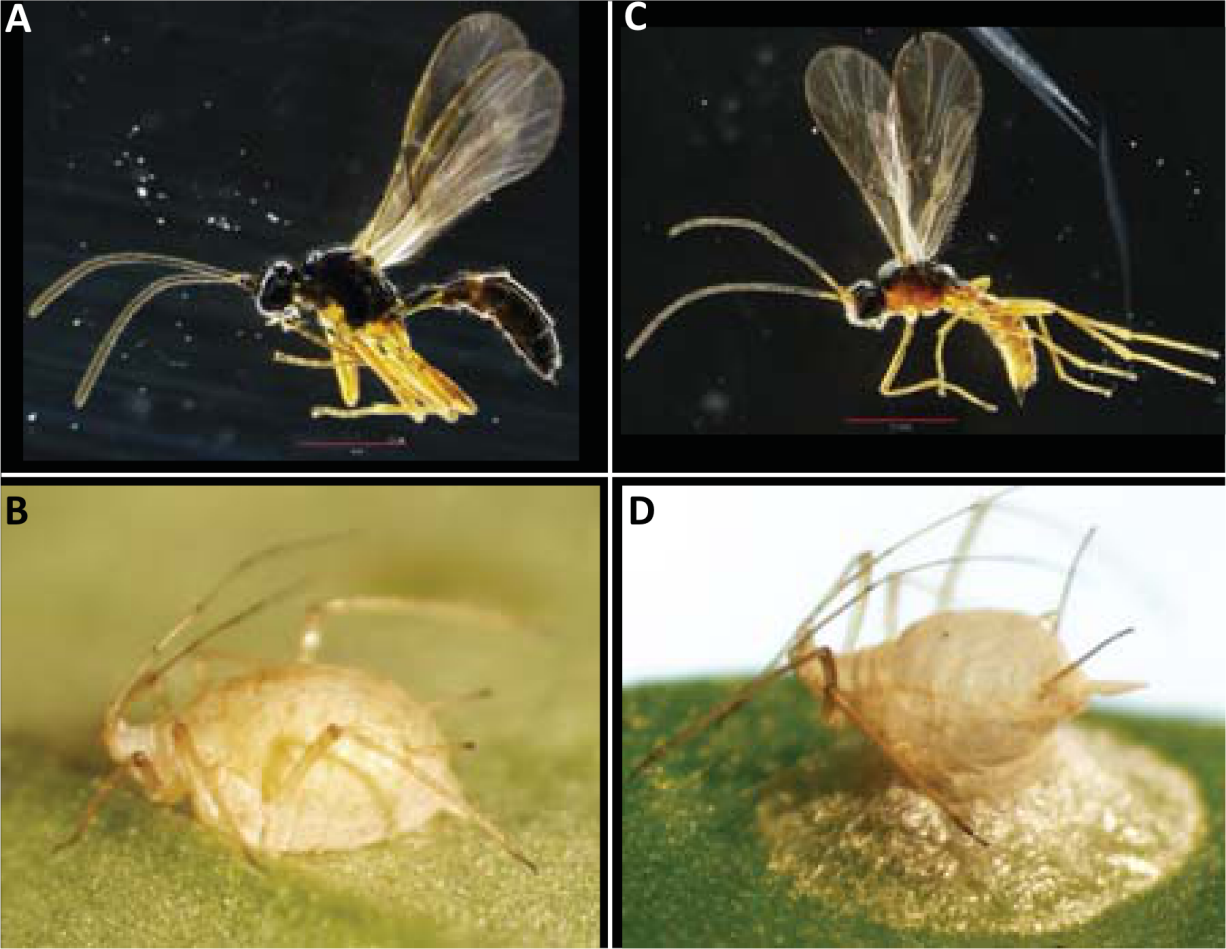
(A,B) Adult female *Aphidius ervi* and aphid mummy. (C,D) Adult female *Praon pequodorum* and aphid mummy.

Since its introduction, *A*. *ervi* has become the dominant parasitoid of North American (N. A. from here on out) pea aphids while the abundance of other, once common, parasitoids have been reduced or completely eliminated [20–23]. For example, another introduced biological control agent, *Aphidius smithi*, as well as the native *A*. *pisivorus* were largely, if not completely, displaced by *A*. *ervi* [20, 33], while the native *Praon pequodorum* (Hymenoptera: Braconidae: Aphidiinae) (Fig 1C and 1D), a once abundant parasitoid of pea aphids, retains viable, although diminished populations [34]. The elimination of parasitoid species other than *A*. *ervi* attacking N. A. pea aphids is likely a function of competitive exclusion [21, 34] and several studies have provided explanations for the persistence of *P*. *pequodorum*. First, experimental bioassays indicated that *P*. *pequodorum* larvae internally outcompeted *A*. *ervi* in instances of multiparasitism within the same host [21, 35], which may happen when aphid populations are low and hosts are scarce [36]. And, second, while *A*. *ervi* may typically be the better external competitor (frequency of host encounter and oviposition), the presence of common non-target aphid species like the spotted alfalfa aphid may reduce the foraging efficiency of *A*. *ervi* more than *P*. *pequodorum* [37].

Another factor potentially contributing to the persistence of *P*. *pequodorum* is that defensive symbionts like *H*. *defensa* or aphid-encoded resistance, may vary in effectiveness or specialize toward particular parasitoid species [13, 18, 38]. Here, we performed parasitism assays on several experimental pea aphid lines to determine whether aphid-encoded and symbiont-mediated resistance vary in levels of protection conferred towards these two wasp species. We also performed serial dissections of parasitized aphids to determine whether there are differences in developmental trajectory of either wasp species that may account for any observed differences in aphid susceptibility after parasitism.

## Materials and Methods

### Pea aphids and creation of experimental lines

The pea aphid, *Acyrthosiphon pisum*, was introduced to North America from Europe in the late 1800s [39–41]. This aphid is cyclically parthenogenetic, reproducing asexually via viviparous production of clonal offspring during the Spring and Summer; sexual morphs arise in the Fall in response to shortening day-lengths [42]. Clonal aphid lines were maintained in the laboratory by rearing them under long day conditions in environmental incubators. Aphid lines used in this study (Table 1) differ in genotype and/or infection status with *H*. *defensa*, and were collected from several different locations. Each line was initiated from a single parthenogenetic female placed onto a caged broad bean plant, *Vicia faba*, and reared at 20±1°C with a 16L: 8D photoperiod. Lines AS3ø, AS3+APSE2, and AS3+APSE3 all share the same aphid genotype but are uninfected with *H*. *defensa*, infected with *H*. *defensa*-APSE2, and infected with *H*. *defensa-*APSE3, respectively. Line AS3+APSE3 is the original aphid line, AS3ø was cured of its *H*. *defensa* infection, as in [8, 43], line AS3+APSE2 is infected with an *H*. *defensa* strain from aphid line ZA17 [8], and was created for use in this study. Aphid genotype CJ113, used in another experiment, was experimentally infected with the same strains as the AS3 genotype above. All experimentally infected/cured aphid lines were established more than 6 months prior to their use in this study. Diagnostic PCR and microsatellite analyses were used to determine symbiont infection status and clonal identity of each aphid line, as in [25].

**Table 1:** Aphid clonal lines used in this study.

Aphid Clonal Line	Secondary symbiont	Collection Location	Reference
WA4ø	uninfected	Pennsylvania 2010	[8, 25]
G15ø	uninfected	Georgia 2008	[44]
*AS3ø	uninfected	Experimentally established	[8, 25]
*AS3+APSE2	*H*. *defensa* + APSE2	Experimentally established	This paper
*AS3+APSE3 (original)	*H*. *defensa* + APSE3	Utah 2007	[8, 25, 26]
WI301-33	*H*. *defensa* + APSE2	Wisconsin 2014	This paper
WI412-52	*H*. *defensa* + APSE3	Wisconsin 2014	This paper
*CJ113ø (original)	uninfected	Utah 2012	[25]
*CJ113+APSE2	*H*. *defensa* + APSE2	Experimentally established	This paper
*CJ113+APSE3	*H*. *defensa* + APSE3	Experimentally established	This paper

Aphid lines sharing the same genotype, but different infection status are indicated with an asterisk; see methods for information on the creation of these experimental lines. Genotype CJ113 aphids were only used for *P*. *pequodorum* fitness assays.

### Parasitoids: *Aphidius ervi* and *Praon pequodorum*

The two most abundant parasitoids currently attacking the pea aphid in N. A. are *Aphidius ervi* and *Praon pequodorum* (Fig 1). Like the pea aphid, *A*. *ervi* is also native to Eurasia, and is a generalist parasitoid of large Macrosiphinae aphids [45–47], though pea aphids appear to be the preferred host [48]. To aid in control of pea aphids, multiple introductions of *A*. *ervi* to N. A. occurred between 1959 and 1968 and this wasp has now established throughout N. A. [20, 33, 49, 50]. Historically more abundant, *P*. *pequodorum* is native to N. A. and its populations on pea aphids saw declines after the introductions of *A*. *ervi* and *A*. *smithi* [21].

*Aphidius ervi* and *P*. *pequodorum* reside within the Aphidiinae subfamily of Braconidae, which is composed of parasitic wasps that attack aphids, but belong to the separate tribes Aphidiini and Praiini, respectively [51–54]. Both are solitary endoparasitoids, typically injecting a single egg into their aphid host at oviposition, which develops to adulthood in the still-living aphid. Mummification of aphids attacked by *A*. *ervi* (Fig 1B), followed by the wasp’s pupation, typically occurs 8–10 days after parasitism at 20°C [55], whereas those attacked by *P*. *pequodorum* mummify (Fig 1D) 6–8 days after parasitism [56]; timing of development was confirmed through personal observation (AJM). Adult wasps of both species eclose approximately five days after mummification. While better studied for *A*. *ervi*, both wasps employ a variety of tactics, including deployment of venom and teratocytes, to overcome aphid defenses and create an environment suitable for wasp development [57, 58].

The *A*. *ervi* wasps (Fig 1A) used in this study were obtained from a single, large, interbreeding laboratory colony containing a mixture of wasps collected from Wisconsin and North Dakota, as well as commercially produced wasps (Arbico Organics). The *P*. *pequodorum* wasps (Fig 1C) were obtained from a single, large, interbreeding laboratory colony containing a mixture of wasps collected from Wisconsin and North Dakota. Wasps were reared continuously on a mixture of the same susceptible aphid lines which were uninfected with facultative symbionts; adults were provided with constant access to honey and water.

### Aphid parasitism resistance assays

We conducted parasitism assays, as in [25, 26], across numerous aphid lines (Table 1) to determine each line’s resistance phenotype against *A*. *ervi* and *P*. *pequodorum*. Briefly, 2^nd^ to 3^rd^ instar aphids (160 total) were singly parasitized (each aphid is removed as it is parasitized). Parasitized aphids were pooled and divided into eight replicates of 20 before being placed on a fresh *V*. *faba* plant in a cup cage and held at 20±1°C and 50% relative humidity with a 16L: 8D photoperiod. Wasps were selected haphazardly from our large laboratory culture and used to singly parasitize individual aphids from each line. Numerous (~ 6–10) individual female wasps were used per treatment and parasitized aphids were pooled before being split up into replicate cohorts of 20. Ten days after parasitism, we counted the number of live aphids, dead aphids, and aphid mummies (dried aphids containing a wasp pupa) to determine the proportion of each, measured as: aphid survival (live aphids/20), dual mortality (both aphid and developing wasp perish/20), and mummification (aphid mummies/20). Mummification rate is used as a suitable and accurate proxy for successful parasitism (instead of wasp emergence rate), as a large majority of wasps emerge after mummification [9]. Aphid mummies were collected from each replicate and were monitored for an additional week to confirm successful eclosion, confirming mummification as a suitable proxy for both *A*. *ervi* and *P*. *pequodorum*. Four replicates of twenty *unparasitized* 2^nd^ to 3^rd^ instar aphids of each line were also placed on fresh plants and used as a control for background mortality measures.

### Aphid serial dissections and wasp development

The egg and larval development of *A*. *ervi* [8, 55, 59] and *P*. *pequodorum* [21, 35, 56] in pea aphids has been described previously, showing some differences in early morphology of the two species. Here, we performed serial dissections on aphid line AS3ø, which is free of facultative symbionts and susceptible to both wasp species, to confirm reported differences in wasp development while controlling for symbiont status and aphid genotype. At least ten dissections of parasitized aphids were performed for each wasp species at each 24h-interval (from 1 to 144h after parasitism) and a representative image was made for each parasitoid species at every interval. All dissections were performed in 60uL of 1X PBS under an Olympus SZX16 stereo microscope. Photos of wasp egg-larval development stages were taken using an AMG EVOS digital inverted microscope.

An additional experiment was performed, examining sub-lethal effects of aphid resistance on *P*. *pequodorum*. These measurements were taken from approximately 50 wasps emerging from each of the following aphid lines AS3ø, CJ113ø, CJ113+APSE2, and CJ113+APSE3, which represent aphids maintaining the least to most resistance against *A*. *ervi*, with the latter two lines having both symbiont- and aphid-based resistance. Lines CJ113+APSE2 and CJ113+APSE3 were experimentally infected and carry the same *H*. *defensa* strains as AS3+APSE2 and AS3+APSE3, respectively, see ‘creation of experimental lines‘ above. Wasps were allowed to complete development but they were killed at adult emergence by freezing at -20°C for 6 hours and then desiccated by drying at 60°C for 24 hours. We then measured wasp dry weight, right-hind tibia length, and sex ratio. Tibia length measurements were performed at 100x magnification and wasp dry weight was measured on a Mettler MT5 microbalance.

### Statistical analyses

Aphid survival, mortality, and mummification (see above) were determined for each replicate of each parasitized aphid line. Using all collected data we performed a Generalized Linear Model (GzLM) with factorial design, using parasitoid species and aphid line to describe effects on aphid survival, mummification, and mortality. We then used a GzLM to compare aphid survival, mummification, and dual mortality (separately for aphids parasitized by either wasp species) among all aphid lines. To further examine effects of *H*. *defensa* strains on aphid resistance to parasitism, we restricted this same analysis to the controlled aphid genotype (AS3, see Table 1). Post hoc pairwise comparisons to test for differences between aphid lines were performed using Tukey’s honestly significant difference (HSD) tests on arcsine transformed aphid survival, mortality, and mummification proportional data. GzLM was also used to compare mortality of parasitized and control (unparasitized) aphids, both within and across lines. Finally, a GzLM was used to compare effects of *H*. *defensa* infection and APSE strain on the outcome of parasitism by either parasitoid species; this analysis was restricted to a control genotype (AS3) of pea aphids, which had been split into three experimental lines. All generalized linear models were performed with a binomial distribution and logit link function; survival, mortality, and mummification data were also mildly overdispersed and so final test values are reported with a quasibinomial adjustment. Because aphid mortality after parasitism may also be tied to differences among aphid lines, linear regression was performed on mean mortality between unparasitized controls and aphids parasitized by either parasitoid species. The mean mortality was natural log transformed to satisfy normality assumptions of the linear regression.

Fitness measures of *P*. *pequodorum* emerging from parasitized aphids included dry weight at emergence, right-hind tibia length, and sex ratio. Dry weight and tibia length were natural log transformed to satisfy normality assumptions. Right-hind tibia length and wasp weight typically correlate with each other as indicators of wasp fitness, so both were compared to each other via linear regression analysis. Analyses of variance (ANOVA) were used to compare dry weight and tibia length (separately for male and female): among four aphid lines, between lines infected/uninfected with *H*. *defensa*, and between *H*. *defensa*-infected lines with APSE2 or APSE3. Finally, we compared sex ratios of emergent wasps using Fisher’s exact test (FET).

## Results

There are three potential outcomes after an aphid is parasitized by either wasp species. 1) *Aphid Survival*: The aphid survives to adulthood and the developing wasp dies. 2) *Mummification*: The developing wasp survives and pupates, mummifies the aphid, and eventually emerges as an adult. 3) *Dual Mortality*: Both aphid and wasp die due to the stresses of this antagonistic interaction, which is a null outcome for both parties involved.

Using a GzLM to describe the interactions between wasp species and experimental aphid lines (Table 2) we found significant effects on all three outcomes owing to differences in wasp species’ ability to overcome aphid defenses and differences among the eight experimental aphid lines in their ability to overcome parasitism.

**Table 2:** Generalized linear model (GzLM) with factorial design, showing effects of wasp species and aphid line on aphid susceptibility to parasitism.

	DF	Aphid Survival	Mummification	Dual Mortality
**Wasp Species**	1	χ2 = 123.9, p < **0.0001**	χ2 = 331.0, p < **0.0001**	χ2 = 0.01, p = 0.9111
**Aphid Line**	7	χ2 = 171.1, p < **0.0001**	χ2 = 176.5, p < **0.0001**	χ2 = 26.0, p = **0.0005**
**Wasp Species X Aphid Line**	7	χ2 = 79.0, p < **0.0001**	χ2 = 156.3, p < **0.0001**	χ2 = 5.5, p = 0.5991
**Whole Model**	15	χ2 = 834.0, p < **0.0001**	χ2 = 678.9, p < **0.0001**	χ2 = 31.3, p = **0.0080**

Significant values indicated in bold.

### Aphid susceptibility to *Aphidius ervi*

**Fig 2:**
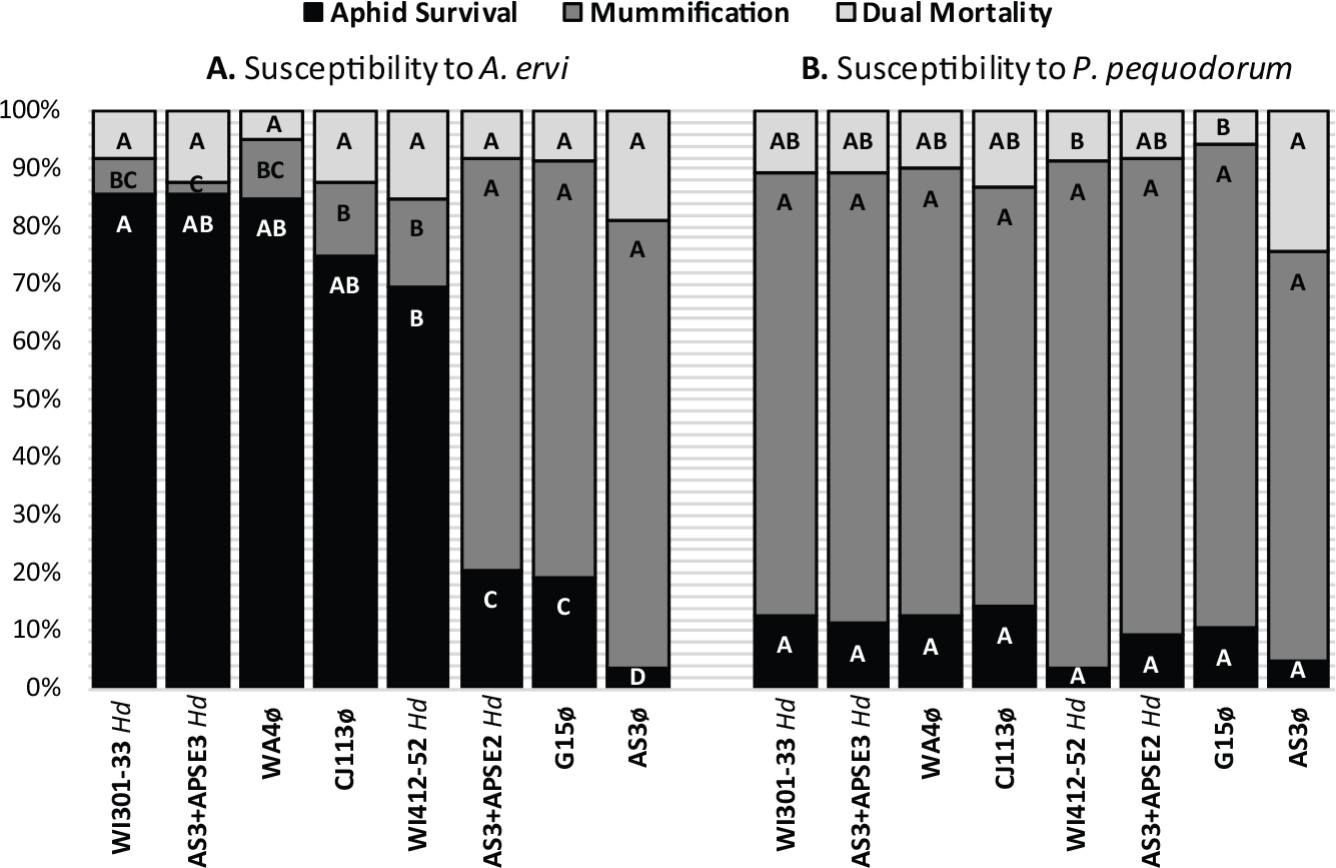
**Pea aphid susceptibility to (A) *Aphidius ervi* and (B) *Praon pequodorum***, measured as mean aphid survival, mummification, and dual mortality. Aphid lines infected with *H*. *defensa* are indicated with ‘*Hd’*. Statistical analyses (GzLMs) were performed separately on aphids parasitized by either wasp species. Significant differences are indicated by letters (Arcsine transformed ANOVA, Tukey’s HSD α  = 0.05). Refer to Table 1 for more information on aphid lines. See the text for GzLM analyses and S1A & S1B Table for arcsine transformed ANOVA.

Overall, we found strongly significant variation in aphid survival and mummification, but no significant variation in dual mortality among the eight pea aphid clonal lines exposed to *A*. *ervi* (Fig 2A). We found significant variation in pea aphid susceptibility to this parasitoid wasp (Aphid Survival: GzLM, χ2 = 422.1, df = 7, p < 0.0001), and aphid survival rates ranged from 4–86%, depending on experimental line. In general, there is an inverse relationship between aphid survival and mummification, and the latter also varied significantly (Mummification: GzLM, χ2 = 396.0, df = 7, p < 0.0001) with rates ranging from 2–72%. We found no significant differences in dual mortality among parasitized aphid lines (Dual Mortality: GzLM, χ2 = 11.5, df = 7, p < 0.1170), which ranged from 8–19%. Our results here were consistent with past studies demonstrating that lines WA4ø and CJ113ø have highly resistant genotypes, while G15ø and AS3ø have highly susceptible genotypes [25]. Similarly, aphids carrying the *H*. *defensa-*APSE3 strain (line AS3+APSE3) were highly resistant to *A*. *ervi*, while those with *H*. *defensa-*APSE2 (line AS3+APSE2) were only moderately resistant [8]. All phage-carrying *H*. *defensa* strains characterized to date confer some protection [60], but since the *H*. *defensa* strains and genotypes of aphid lines WI301-33 and WI412-52 (Table 1) have not been experimentally partitioned it is unclear how much protection is attributable to each potential source for these two lines.

### Aphid susceptibility to *Praon pequodorum*

In contrast to our results with *A*. *ervi*, we found no significant variation in aphid survival and mummification across our eight experimental aphid lines following exposure to *P*. *pequodorum* (Fig 2B); aphid survival ranged from 4–14% (Aphid survival: GzLM, χ2 = 12.4, df = 7, p < 0.0892) while mummification ranged from 71–88% (Mummification: GzLM, χ2 = 11.3, df = 7, p < 0.1255). Thus, despite the inclusion of a range of aphid genotypes and *H*. *defensa* strains that exhibit strong variation in resistance to *A*. *ervi*, we found that, surprisingly, all experimental aphid lines utilized in this study were uniformly highly susceptible to parasitism by *P*. *pequodorum*. We did detect significant variation in mortality among the eight pea aphid clonal lines exposed to *P*. *pequodorum* (Dual mortality: GzLM, χ2 = 20.9, df = 7, p < 0.0039), which is largely attributable to a single line, AS3ø. However, the control unparasitized AS3ø line also shows higher mortality than other control lines (see S1 Fig) so it does not appear that this mortality is specifically due to parasitism by *P*. *pequodorum*.

### Analysis of aphid mortality

Occasionally, neither parasitoid nor host aphid survive the parasitism event, resulting in dual mortality; here we compared the mortality of unparasitized control lines with the dual-mortality present in parasitized lines to establish whether parasitism resulted in mortality rates above background. We found aphid mortality to be significantly variable across control lines not exposed to wasps (GzLM, χ^2^ = 15.2, df = 7, p = 0.0330), but within lines, parasitism by either wasp species did not result in significant increases in aphid-wasp dual mortality relative to mortality of unparasitized control aphids of the same clonal line (S1 Fig). Across lines, we also found no correlation between mortality of unparasitized control aphids and dual mortality of those parasitized by either *A*. *ervi* (Linear regression, F_1,7_ = 1.73, p = 0.2368) or *P*. *pequodorum* (Linear regression, F_1,7_ = 0.64, p = 0.4551), meaning that aphid-wasp dual mortality is affected differently by parasitism and does not trend with the background mortality levels of aphid lines.

### Restricted analysis to single aphid genotype

**Fig 3:**
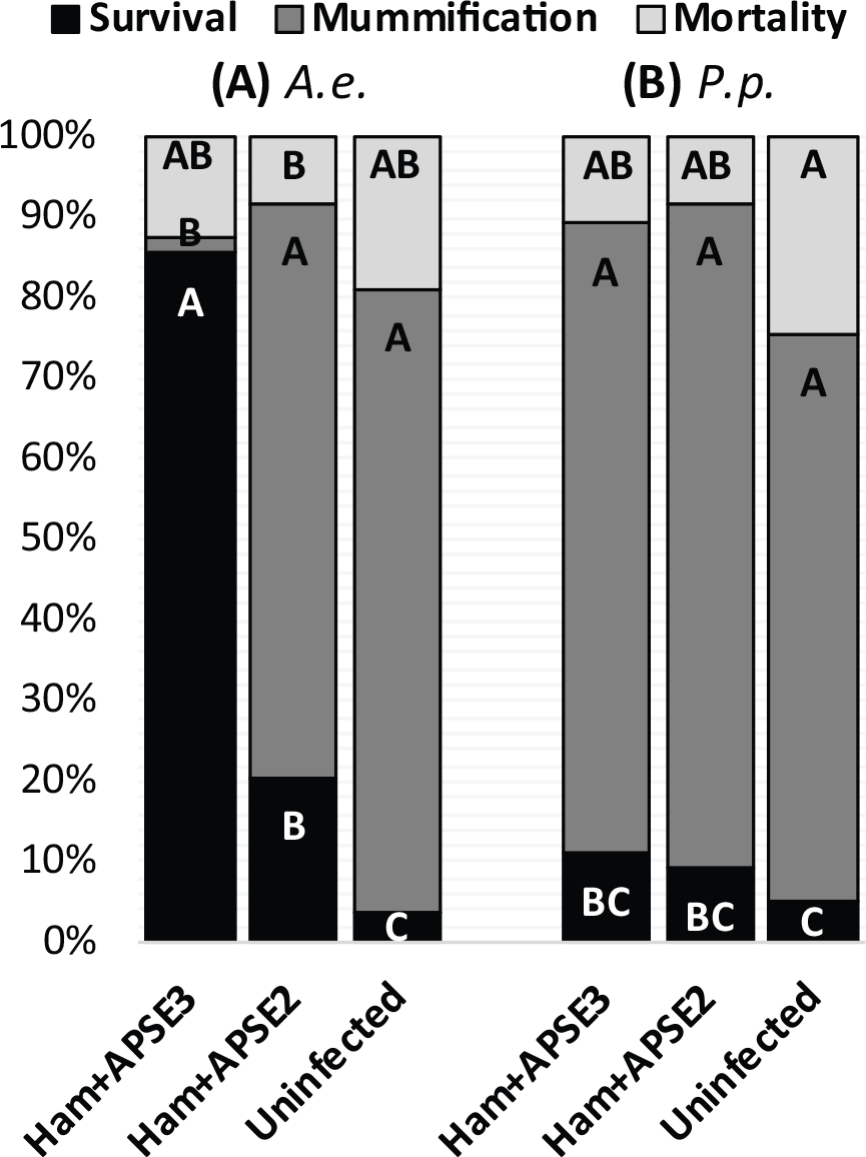
**Effect of *H*. *defensa* and APSE strain on aphid susceptibility to (A) *Aphidius ervi* and (B) *Praon pequodorum*.** The three aphid clonal lines used here are of a single genotype (AS3) and are uninfected with *H*. *defensa* (line AS3ø), infected with *H*. *defensa+*APSE2 (line AS3+APSE2), and infected with *H*. *defensa*+APSE3 (line AS3+APSE3). P < 0.0001, <0.0001, = 0.0078 for survival, mummification, and mortality, respectively (GzLM). Letters indicate significant differences (Arcsine transformed Tukey’s HSD α  = 0.05). See ‘Table 3‘ for GzLM effect tests of *H*. *defensa* infection and APSE strain on aphid susceptibility.

We also conducted analyses restricted to lines where the aphid genotype (AS3) was held constant, but varied in presence (+/-) and type of defensive symbiont (AS3ø, AS3+APSE2, or AS3+APSE3, Table 1) to determine conclusively whether the two most common strains of *H*. *defensa* in N. A. confer protection to *P*. *pequodorum*. As shown previously, we find significant effects of *H*. *defensa* infection on successful parasitism by *A*. *ervi* (infected aphids have higher survival, lower mummification) and APSE strain correlated with strength of protection (APSE3 provides stronger protection than APSE2; Fig 3, Table 3) and no significant effects were observed on aphid mortality. In contrast, *H*. *defensa* infection with either APSE2 or APSE3 had no effects on aphid survival or mummification after parasitism by *P*. *pequodorum* compared to genetically identical, uninfected controls. Mortality was significantly affected by *H*. *defensa* infection due to the uninfected line, AS3ø, having higher mortality than either of the *H*. *defensa* infected lines. Thus, the protective effects of *H*. *defensa* appear specialized to the dominant parasitoid *A*. *ervi* and have no significant effects on *P*. *pequodorum*.

**Table 3:** Generalized linear model showing effects of ‘protective’ symbiont infection and infecting bacteriophage APSE strain on aphid susceptibility to parasitism.

	Explanatory Variable	DF	Aphid Survival	Mummification	Mortality
***A*. *ervi***	*H*. *defensa* infection	1	χ2 = 19.8, p < **0.0001**	χ2 = 8.44, p = **0.0037**	χ2 = 3.15, p = 0.0759
** **	APSE Strain	1	χ2 = 86.9, p < **0.0001**	χ2 = 217.3, p < **0.0001**	χ2 = 0.95, p = 0.3305
***P*. *pequodorum***	*H*. *defensa* infection	1	χ2 = 3.01, p = 0.0828	χ2 = 3.54, p = 0.0598	χ2 = 14.3, p = **0.0002**
** **	APSE Strain	1	χ2 = 0.17, p = 0.6786	χ2 = 0.77, p = 0.3788	χ2 = 0.64, p = 0.4238

Analyses were performed separately on aphids parasitized by either wasp species and were restricted to aphid sublines within the AS3 genotype (see Fig 3). Significant values indicated in bold.

### Variations in wasp development may underlie differences in aphid susceptibility

**Fig 4:**
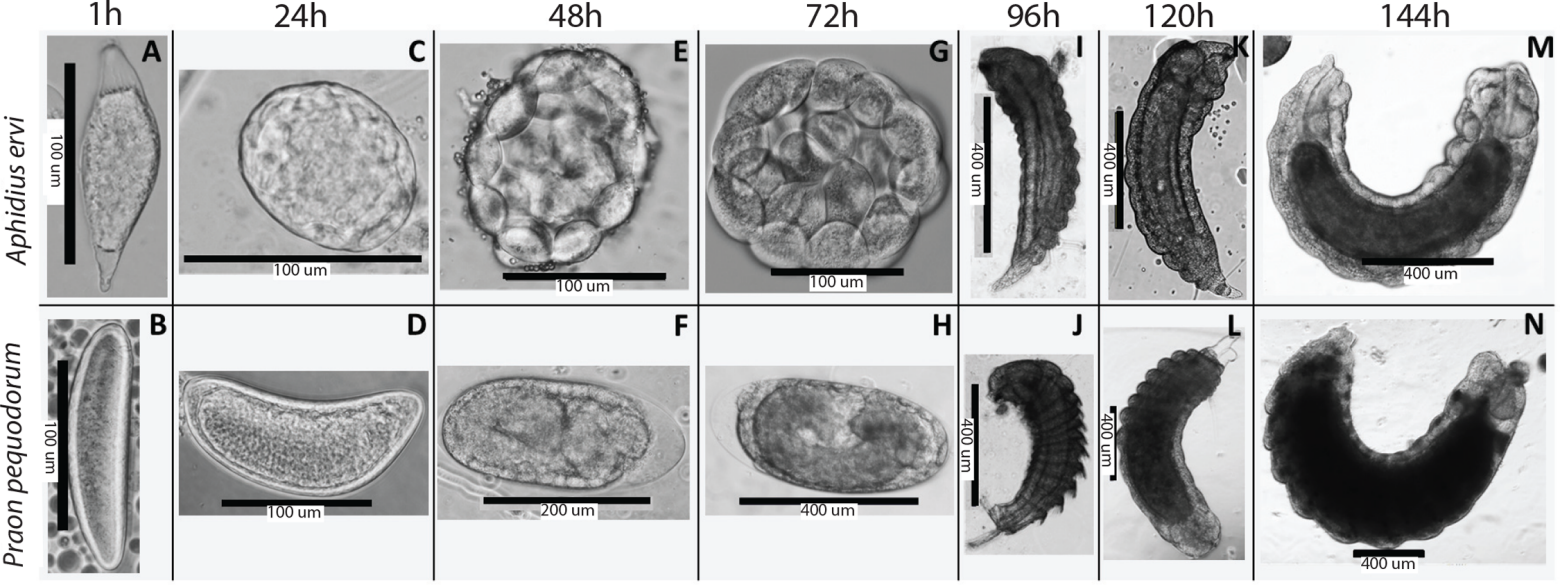
Serial dissections of parasitized pea aphids, revealing differences in early parasitoid wasp development. Development of *Aphidius ervi* (top) and *Praon pequodorum* (bottom). (A and B) Eggs of both parasitoids 1 hour after parasitism. (C) The morula of *A*. *ervi* shortly after the egg has hatched, revealing the developing embryo surrounded by serosal cells. (D) The egg of *P*. *pequodorum* remains intact with a thick chorion. (E) The *A*. *ervi* morula continues to grow. (F) The *P*. *pequodorum* egg has not yet hatched, but continues to grow. (G) First instar *A*. *ervi* larva (not shown) emerges from morula, serosal cells have not yet dissociated to form teratocyte cells. (H) The egg chorion, serosal cells, and developing embryo of *P*. *pequodorum* are clearly visible. The egg finally hatches between 72–96h. (I and J) Second instar larvae of both wasps. (K-N) Similar continued larval development of both wasps.

Serial dissections of parasitized aphids at 24h intervals confirmed that while the overall developmental trajectories of *A*. *ervi* and *P*. *pequodorum* are similar (Fig 4) some striking differences exist through the 72h time-point, namely that the chorion of the *A*. *ervi* egg ruptures around 24h, potentially exposing the wasp embryo to aphid or symbiont defenses, whereas the *P*. *pequodorum* egg chorion does not rupture until after 72h. Mortality to *A*. *ervi* wasps in highly resistant aphids typically occurs prior to 72h [8].

At 1h, we found that *A*. *ervi*’s egg is an elongate oval with tapered ends and each end is translucent (Fig 4A); we find the chorion here to be thinner than that of *P*. *pequodorum* (Fig 4B). The egg chorion then ruptures around 24h releasing a roughly spherical morula, composed of the growing serosal teratocyte cells which surround the developing wasp (Fig 4C). At 48h the morula is roughly spherical (Fig 4E). At 72h the morula has increased in size and is roughly spherical; shortly thereafter, the larval parasitoid emerges from the morula (Fig 4G). The *A*. *ervi* larva continues its growth through the last measurement at 144h (Fig 4M).

At 1h the *P*. *pequodorum* egg is an opaque elongate oval with rounded ends (Fig 4B). At 24h the elongate egg and a distinct thickened chorion (compared to *A*. *ervi*) are still intact (Fig 4D). At 48h the egg and chorion remain intact and some, but not all, eggs have a distinct translucent portion of the chorion at one end (Fig 4F). At 72h the egg and chorion still remain intact (Fig 4H) and the first instar wasp larva emerges from the egg between 72h and 96h. The *P*. *pequodorum* larva continues growing through the last measurement at 144h (Fig 4N).

### Sub-lethal fitness effects of resistance on *P*. *pequodorum*

Though *P*. *pequodorum* is able to consistently develop in aphid lines resistant to *A*. *ervi*, we tested whether there were sub-lethal fitness effects on *P*. *pequodorum* emerging from aphids maintaining both endogenous *and H*. *defensa*-mediated resistance against *A*. *ervi* relative to control lines susceptible to *A*. *ervi*. Comparing wasps emerging from the most susceptible aphid genotype (AS3) to one of the most resistant (CJ113), which we also experimentally infected with protective *H*. *defensa* strains, maximized the likelihood of detecting sub-lethal costs of either type of resistance on fitness, if any. As indicators of *P*. *pequodorum* fitness we measured dry weight at emergence and right-hind tibia length for both males (♂) and females (♀). As expected, we found that dry weight and tibia length were strongly correlated with each other among both males and females (Linear regression, R^2^ = 0.70 ♂ / 0.77 ♀ F = 180.6 ♂ / 312.8 ♀, p < 0.0001 ♂ and ♀). Among female wasps, *P*. *pequodorum* dry weight and tibia length did not vary significantly with respect to the resistance phenotype (i.e. between the susceptible AS3 and resistant CJ113 lines), *H*. *defensa* or APSE strain types (Table 4A, S2 Fig). Male wasp dry weight varied significantly by resistance phenotype (susceptible 0.131mg, resistant 0.116mg) and male tibia length varied significantly among aphid lines (AS3ø 763.5um, CJ113ø 739.0um, CJ113+APSE2 729.8um, CJ113+APSE3 791.4um) and between APSE types (CJ113+APSE2 729.8um, CJ113+APSE3 791.4um), but each’s corresponding tibia length and dry weights, respectively, did not follow the same pattern (Table 4A, S2 Fig). We also measured sex ratio of *P*. *pequodorum* but found they did not vary by aphid line, aphid resistance phenotype, *H*. *defensa* infection, or APSE strain (Table 4B).

**Table 4:** Fitness measures of *P*. *pequodorum* wasps emerging from aphid lines AS3ø, CJ113ø, CJ113+APSE2, and CJ113+APSE3.

**A.**			**Male wasps (♂)**			**Female wasps (♀)**		
**Variable**	**Fitness measure**	**df**	**n**	**f-val.**	**p-val.**	**n**	**f-val.**	**p-val.**
Aphid Line	Dry weight	3	77	2.47	0.0687	96	1.55	0.2061
	Tibial length	3	77	5.67	**0.0015**	96	1.85	0.1431
Aphid Genotype	Dry weight	1	77	3.98	**0.0496**	96	1.35	0.2489
	Tibial length	1	77	0.77	0.3841	96	0.45	0.5039
*H*. *defensa* infection	Dry weight	1	77	1.20	0.2765	96	0.01	0.9222
	Tibial length	1	77	0.21	0.6468	96	0.35	0.5545
APSE strain	Dry weight	1	36	2.26	0.1422	51	2.85	0.0978
	Tibial length	1	36	10.52	**0.0026**	51	3.71	0.0599
**B.**		**n ♂**	**n ♀**	**p-val.**				
Aphid line	AS3ø	21	21	0.4975				
	CJ113ø	20	24					
	CJ113+APSE2	19	20					
	CJ113+APSE3	17	31					
Aphid genotype	AS3 (susceptible)	21	21	0.4765				
	CJ113 (resistant)	56	75					
*H*. *defensa* infection	Infected	36	51	0.4461				
* *	Uninfected	41	45					
APSE strain	APSE2	19	20	0.2746				
	APSE3	17	31					

(A) Analysis of dry weight and right-hind tibia length of emergent wasps (ANOVA). (B) Sex ratios of emergent wasps (Fisher’s exact test). See S2 Fig for dry weight and tibia length values.

## Discussion

As expected from previous studies, we found substantial variation in pea aphid- and *H*.*defensa*/APSE-sourced resistance to *A*. *ervi*, the dominant parasitoid of this aphid in N. A. (Fig 2A) [25, 26, 30]. Using these same lines that varied in aphid- and symbiont-based resistance to *A*. *ervi*, however, we found no variation in pea aphid susceptibility to the related, and second most abundant parasitoid, *P*. *pequodorum*, (Fig 2B). Further, we show that *H*. *defensa* strains carrying either APSE2 or APSE3 confer no resistance to *P*. *pequodorum* relative to uninfected controls sharing the same aphid genotype (Fig 3, Table 3). Similarly, all pea aphid genotypes (uninfected with symbionts) that were resistant to *A*. *ervi* were highly susceptible to *P*. *pequodorum*. Not only were *P*. *pequodorum* able to develop in all aphid lines resistant to *A*. *ervi*, we found no consistent sub-lethal fitness costs to *P*. *pequodorum* developing in resistant lines.

Such specificity of *H*. *defensa* to particular natural enemies has now been shown in all three cases where this symbiont is known to confer protection (pea aphid, cowpea aphid, and black bean aphid) [13, 18]. *H*. *defensa* in cowpea aphid, *Aphis craccivora*, conferred protection against *Binodoxys communis* and *B*. *koreanus*, but not against *Lysiphlebus orientalis* or *Aphidius colemani* (all Hymenoptera: Braconidae: Aphidiinae) [13] while *H*. *defensa* in the black bean aphid, *Aphis fabae*, protected against *L*. *fabarum* and *A*. *colemani*, but not against *B*. *angelicae* or *Aphelinus chaonia* [18]. Most recently, *H*. *defensa* strains from several European pea aphid biotypes were shown to vary in levels of resistance conferred to *A*. *ervi* versus *Aphelinus abdominalis*, a distantly related parasitoid (Chalcidoidea; Aphelinidae) [17], although we note that this parasitoid is not commonly found in N. A. populations. Together these findings indicate that specificity of protective *H*. *defensa* to particular natural enemies is likely a general phenomenon.

In pea aphids, both the aphid-encoded and symbiont-based defenses tested provided specific and effective protection against *A*. *ervi*, but had no effect on *P*. *pequodorum*. It remains possible that uncommon, and hitherto undiscovered strains of *H*. *defensa* are protective against *P*. *pequodorum*. However, given that the *H*. *defensa* strains examined contained the dominant APSE types (2 & 3) present in N. A. pea aphid populations, this indicates that it is likely that *H*. *defensa* is not generally effective against *P*. *pequodorum*. Similarly, if we sampled additional aphid genotypes we may discover some that are resistant to this wasp.

Aphids that are resistant to *A*. *ervi* may serve as a reservoir of hosts available only to *P*. *pequodorum* and, at least partially, explain why this wasp has not been completely eliminated by competition, as is the case with *A*. *smithi* and other previously common parasitoids [20–23]. Before the introduction of *A*. *ervi*, *P*. *pequodorum* was relatively abundant, making up 42% of parasitized pea aphids on alfalfa in Wisconsin, USA [61] and 25% in British Columbia, Canada [62]. Since *A*. *ervi*’s introduction, though, populations of *P*. *pequodorum* have steadily declined with more recent estimates ranging between 6% in British Columbia [21], 8% (or locally absent) in Wisconsin [34], and 14% in NY and PA [63]. In contrast, populations of *A*. *ervi* increased to 86–100% of parasitized aphids over the same time-period [21, 63, 64]. Helping to explain *A*. *ervi*’s abundance, these studies found that *A*. *ervi* is more efficient at foraging for aphid hosts than other pea aphid parasitoids, an attribute which may further benefit recolonization of this parasitoid when faced with human agricultural practices such as cutting and harvesting of alfalfa [34]. The persistence of *P*. *pequodorum* in pea aphid populations, however, indicates that it may be able to successfully compete with *A*. *ervi* under certain conditions. Larval competition assays, for example, found that *P*. *pequodorum* consistently outcompeted *A*. *ervi* in instances of multiparasitism [21], which may occur when parasitism rates are high or when aphid populations are low [36]. Further, *A*. *ervi*’s foraging efficiency may be more affected by non-target aphid species than *P*. *pequodorum*’s [37]. Our results indicate that specificity of resistance to *A*. *ervi* is likely another factor contributing to the persistence of *P*. *pequodorum*. Thus, even though *A*. *ervi* is more efficient at foraging for hosts, *P*. *pequodorum* may be able to persist in aphid populations with high *H*. *defensa* infection frequencies or high genotypic resistance to *A*. *ervi*.

More generally, given that many insect hosts are attacked by multiple parasitoids, the invasion of enemy-specific protective symbionts or resistant alleles [65–67], could alter the competition dynamics between parasitoid species, e.g. [68–70]. Common insect symbionts with known roles in defense like *Wolbachia* and *Spiroplasma* can spread rapidly through insect populations [71–74] and are also shown to vary temporally and spatially, e.g. [75], potentially resulting in rapid changes in the composition of natural enemies. Future field or population cage studies could, for example, show that high levels of *H*. *defensa* or resistant genotypes result in increases in the abundance of *P*. *pequodorum* at the expense of *A*. *ervi*. On the other hand, differences in natural enemy ability to overcome symbiont-based defenses may, in turn, influence composition and frequency of symbiont infections in the field. Pea aphids infected with *H*. *defensa* only occur at intermediate frequencies throughout natural populations of pea aphids [75], but with such clear advantages to infection in the face of parasitism by *A*. *ervi* and near 100% vertical transmission efficiency in laboratory colonies, it may be surprising that a higher proportion of aphids are not infected. Selective factors that may limit its spread include infection costs in the absence of parasitism [76, 77], the presence of resistant uninfected genotypes [25], or ineffectiveness under specific environmental conditions [78] or against other natural enemies, such as *P*. *pequodorum*.

Further work is needed to understand the basis for differential resistance to *A*. *ervi* vs. *P*. *pequodorum*. It is possible that aphid and symbiont encoded factors specifically target *A*. *ervi* genotypes, or it may be that *P*. *pequodorum* exhibits features that counter or prevent effective aphid defenses. For example, because *A*. *ervi* and the pea aphid originated in Eurasia and share a long evolutionary history, pea aphid defense mechanisms may have evolved specifically to combat *A*. *ervi* and other common native Eurasian parasitoids. We did, however, find marked differences in early egg-larval development between these two wasp species (Fig 4) that may be involved with the observed differences in aphid resistance. While the overall biology and development of both wasps is similar, we found that the thinly chorionated *A*. *ervi* egg hatches around 24 hours after parasitism while the thicker chorion of the *P*. *pequodorum* egg remains intact until after 72 hours. This is important because the highly-resistant *H*. *defensa-*APSE3 strain causes mortality to *A*. *ervi* between 24–48 hours after parasitism, after the chorion has ruptured, but before first instar larva develops. Hence, prolonged development in a chorionated egg may protect developing *P*. *pequodorum* during the stages which are most susceptible to symbiont-incurred damage.

Given that *H*. *defensa* confers variable resistance against a range of aphid parasitoid species, comparative developmental studies among wasp species could reveal whether prolonged sequestration of the embryo inside a protective chorion is a general strategy to circumvent symbiont-mediated defense. However, such a strategy is clearly not universal. First, in black bean aphids, *H*. *defensa* protection is not only specific to particular wasp species, but also to particular genotypes within wasp species [18, 79, 80]. Second, in *A*. *pisum*, mortality to *A*. *ervi* owing to both aphid-encoded and *H*. *defensa* strains with APSE2 often occurs after this 72h window, and *P*. *pequodorum*, whose larva is usually exposed by this point, is just as successful at attacking pea aphid with these types of resistance to *A*. *ervi*. And finally, *H*. *defensa* confers protection against *A*. *colemani* in black bean aphids, but not in cowpea aphids [13, 18], although developmental studies show that *A*. *colemani* emerges from its egg as late as two days after parasitism [81] so it possible that this is important in cowpea aphid, but not black bean aphid due to the timing of symbiont-induced harm. Clearly, more work is needed to understand mechanisms underlying the specificity of parasitoid resistance.

## Conclusions

Here we show that multiple resistance mechanisms that pea aphids use to combat their most abundant parasitoid *A*. *ervi*, including protective bacterial symbionts and resistant genotypes, are ineffective against a related, but less common parasitoid, *P*. *pequodorum*. Given that *A*. *ervi* is a superior external competitor, pea aphid resistance to *A*. *ervi* may be one mechanism, which allows *P*. *pequodorum* to persist in North American populations of pea aphids, where all other parasitoids have been eliminated. However, in a more evenly matched competitive interaction, the introduction of resistance could potentially give a strong selective advantage of one natural enemy over another, which could lead to dramatic shifts in natural enemy composition. Nevertheless, our findings suggest that host-symbiont infections and endogenous resistance are important not only to their ecology, but the ecology of their competing natural enemies, potentially influencing the guilds of natural enemies attacking a host species in a given area.

## Supporting Information

S1 Fig Comparison of mortality (not resulting in mummification) among aphid lines. (PDF)

S2 Fig Fitness measures of adult *P*. *pequodorum* emerging from susceptible (AS3) and resistant (CJ113) aphid lines. (PDF)

S1 Table Arcsine transformed ANOVAS of aphid susceptibility to parasitism. (PDF)
