# Supplementary material for: Specificity of Multi-Modal Aphid Defenses against Two Rival Parasitoids
Source: PLoS One. 2016 May 2;11(5):e0154670. doi: 10.1371/journal.pone.0154670 (PMC4852904; doi:10.1371/journal.pone.0154670)
Supplement: S1 Fig — (PDF) [file pone.0154670.s001.pdf]

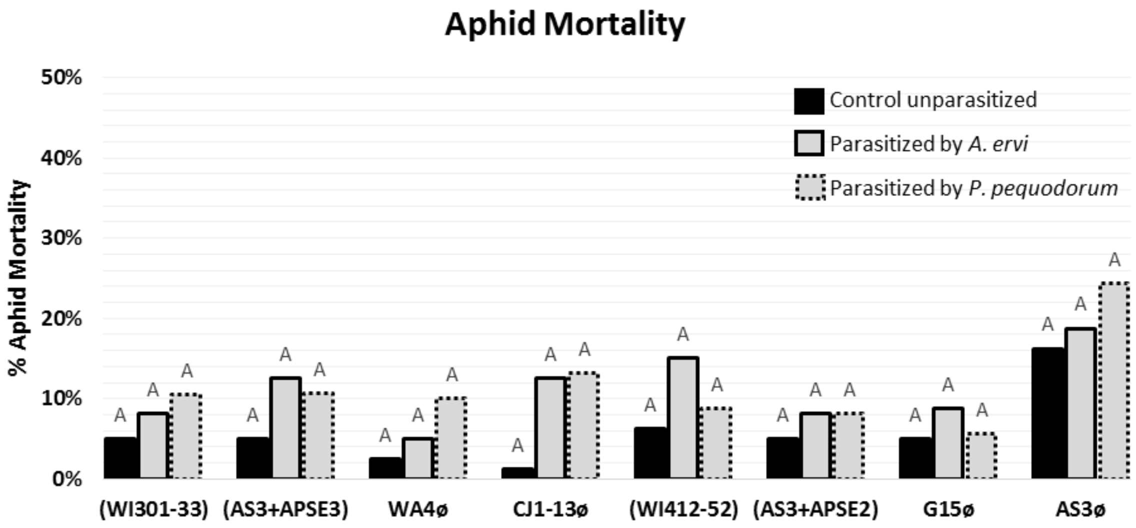

S1 Fig. Comparison of mortality (not resulting in mummification) among aphid lines. Overall significance: p = 0.0002 (GzLM df = 23, $\chi^2 = 55.8$); Control significance: p = 0.0330 (GzLM df = 7, $\chi^2 = 15.2$); see main text and Figure 2 for analyses involving dual mortality due to *A. ervi* and *P. pequodorum* parasitism. Letters indicate lack of significant differences among treatments, within aphid lines (Arcsine transformed ANOVA, Tukey's HSD $\alpha = 0.05$). See supplemental table 1c for arcsine transformed ANOVA.
