# Supplementary material for: Specificity of Multi-Modal Aphid Defenses against Two Rival Parasitoids
Source: PLoS One. 2016 May 2;11(5):e0154670. doi: 10.1371/journal.pone.0154670 (PMC4852904; doi:10.1371/journal.pone.0154670)
Supplement: S2 Fig — (PDF) [file pone.0154670.s002.pdf]

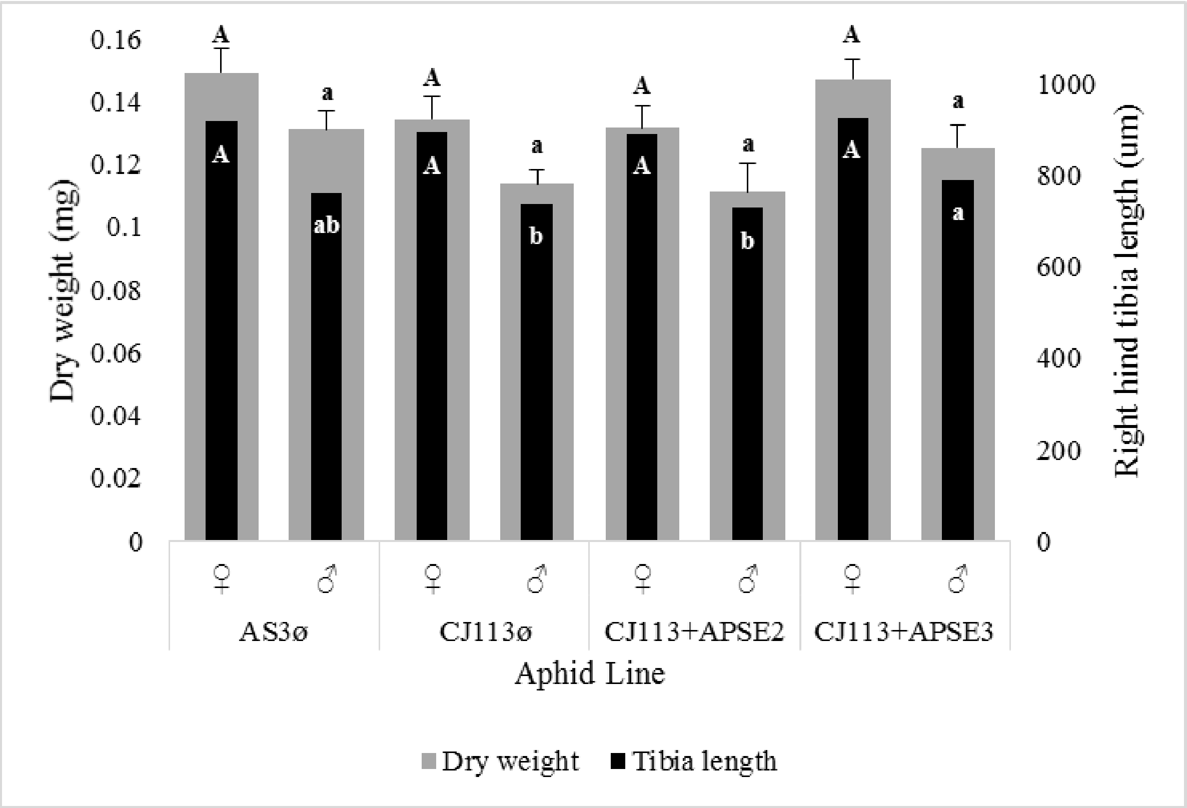

S2 Fig. Fitness measures of adult *P. pequodorum* emerging from susceptible (AS3) and resistant (CJ113) aphid lines. Dry weight, p = 0.2061♀ / 0.0687♂. Tibial length, p = 0.1431♀ / 0.0015♂ (ANOVA). Letters indicate significance among females (capital letters) and among males (lowercase letters) (Tukey's HSD α = 0.05). Note: Error bars are for dry weight only.
