# Supplementary material for: Specificity of Multi-Modal Aphid Defenses against Two Rival Parasitoids
Source: PLoS One. 2016 May 2;11(5):e0154670. doi: 10.1371/journal.pone.0154670 (PMC4852904; doi:10.1371/journal.pone.0154670)
Supplement: S1 Table — (PDF) [file pone.0154670.s003.pdf]

Note: The untransformed means are shown in figures 2 and 3 in the main text and Supplemental figure 1.

Arcsine Transformed	Aphid line	n	Mean	Std. Dev.	Std. Err. Mean.	Lower 95%	Upper 95%
**Survival**	WI301-33	8	1.24809	0.25624	0.09059	1.0339	1.4623
	AS3+APSE3	8	1.19465	0.118803	0.042	1.0953	1.294
	WA4ø	8	1.18138	0.096499	0.03412	1.1007	1.2621
	CJ113ø	8	1.05647	0.143278	0.05066	0.9367	1.1763
	WI412-52	8	0.98831	0.109128	0.03858	0.8971	1.0795
	AS3+APSE2	8	0.44982	0.179868	0.06359	0.2994	0.6002
	G15ø	8	0.45142	0.079897	0.02825	0.3846	0.5182
	AS3ø	8	0.16914	0.104392	0.03691	0.0819	0.2564
**Mummification**	WI301-33	8	0.19477	0.177378	0.06271	0.0465	0.3431
	AS3+APSE3	8	0.08457	0.116714	0.04126	-0.013	0.1821
	WA4ø	8	0.26972	0.196598	0.06951	0.1054	0.4341
	CJ113ø	8	0.34638	0.123487	0.04366	0.2431	0.4496
	WI412-52	8	0.36913	0.198403	0.07015	0.2033	0.535
	AS3+APSE2	8	1.01001	0.116608	0.04123	0.9125	1.1075
	G15ø	8	1.01435	0.078175	0.02764	0.949	1.0797
	AS3ø	8	1.11856	0.24305	0.08593	0.9154	1.3218
**Dual Mortality**	WI301-33	8	0.21595	0.219235	0.07751	0.0327	0.3992
	AS3+APSE3	8	0.33271	0.158403	0.056	0.2003	0.4651
	WA4ø	8	0.1745	0.15614	0.0552	0.044	0.305
	CJ113ø	8	0.30871	0.213775	0.07558	0.13	0.4874
	WI412-52	8	0.37072	0.161536	0.05711	0.2357	0.5058
	AS3+APSE2	8	0.22	0.209472	0.07406	0.0449	0.3951
	G15ø	8	0.25651	0.172447	0.06097	0.1123	0.4007
	AS3ø	8	0.40376	0.231232	0.08175	0.2104	0.5971

	df	F-value	P-value
Survival	7	65.7556	<.0001
Mummification	7	52.4677	<.0001
Dual mortality	7	1.4282	0.2125

S1a Table. Arcsine transformed ANOVA of aphid susceptibility to *Aphidius ervi*.

Arcsine Transformed	Aphid line	n	Mean	Std. Dev.	Std. Err. Mean.	Lower 95%	Upper 95%
**Survival**	WI301-33	9	0.338818	0.153438	0.05115	0.22087	0.45676
	AS3+APSE3	8	0.281726	0.222251	0.07858	0.09592	0.46753
	WA4ø	8	0.350153	0.106043	0.03749	0.2615	0.43881
	CJ113ø	8	0.3562	0.177926	0.06291	0.20745	0.50495
	WI412-52	8	0.13428	0.154219	0.05452	0.00535	0.26321
	AS3+APSE2	8	0.303399	0.080379	0.02842	0.2362	0.3706
	G15ø	8	0.248184	0.253324	0.08956	0.0364	0.45997
	AS3ø	8	0.193194	0.126423	0.0447	0.0875	0.29889
**Mummification**	WI301-33	9	1.072141	0.099134	0.03304	0.99594	1.14834
	AS3+APSE3	8	1.09635	0.140252	0.04959	0.9791	1.2136
	WA4ø	8	1.087558	0.137619	0.04866	0.97251	1.20261
	CJ113ø	8	1.032686	0.162034	0.05729	0.89722	1.16815
	WI412-52	8	1.24718	0.187018	0.06612	1.09083	1.40353
	AS3+APSE2	8	1.151146	0.123336	0.04361	1.04803	1.25426
	G15ø	8	1.226156	0.280197	0.09906	0.99191	1.46041
	AS3ø	8	1.009245	0.169432	0.0599	0.8676	1.15089
**Dual Mortality**	WI301-33	9	0.304586	0.143802	0.04793	0.19405	0.41512
	AS3+APSE3	8	0.324922	0.079593	0.02814	0.25838	0.39146
	WA4ø	8	0.284355	0.17608	0.06225	0.13715	0.43156
	CJ113ø	8	0.338951	0.170478	0.06027	0.19643	0.48147
	WI412-52	8	0.234032	0.208503	0.07372	0.05972	0.40834
	AS3+APSE2	8	0.244484	0.170592	0.06031	0.10187	0.3871
	G15ø	8	0.186528	0.164135	0.05803	0.04931	0.32375
	AS3ø	8	0.498296	0.178005	0.06293	0.34948	0.64711

	df	F-value	P-value
Survival	7	1.8184	0.1013
Mummification	7	2.0828	0.0601
Dual mortality	7	2.6081	0.0209

S1b Table. Arcsine transformed ANOVA of aphid susceptibility to *Praon pequodorum*.

Arcsine Transformed	Aphid line	n	Mean	Std. Dev.	Std. Err. Mean.	Lower 95%	Upper 95%
**Control lines mortality**	WI301-33	4	0.193194	0.136553	0.06828	-0.0241	0.41048
	AS3+APSE3	4	0.193194	0.136553	0.06828	-0.0241	0.41048
	WA4ø	4	0.080438	0.160875	0.08044	-0.1756	0.33643
	CJ113ø	4	0.056378	0.112757	0.05638	-0.123	0.2358
	WI412-52	4	0.249573	0.048119	0.02406	0.173	0.32614
	AS3+APSE2	4	0.193194	0.136553	0.06828	-0.0241	0.41048
	G15ø	4	0.193194	0.136553	0.06828	-0.0241	0.41048
	AS3ø	4	0.354612	0.268482	0.13424	-0.0726	0.78183

	df	F-value	P-value
Control Mortality	7	1.4754	0.2231

S1c Table. Arcsine transformed ANOVA of aphid mortality in the unparasitized control lines.
